# Regulators of homologous recombination repair as novel targets for cancer treatment

**DOI:** 10.3389/fgene.2015.00096

**Published:** 2015-03-20

**Authors:** Małgorzata Krajewska, Rudolf S. N. Fehrmann, Elisabeth G. E. de Vries, Marcel A. T. M. van Vugt

**Affiliations:** Department of Medical Oncology, Cancer Research Center Groningen, University Medical Center Groningen, University of GroningenGroningen, Netherlands

**Keywords:** recombination, Cell Cycle, genomic instability, DNA Repair, PARP inhibitors

## Abstract

To cope with DNA damage, cells possess a complex signaling network called the ‘DNA damage response’, which coordinates cell cycle control with DNA repair. The importance of this network is underscored by the cancer predisposition that frequently goes along with hereditary mutations in DNA repair genes. One especially important DNA repair pathway in this respect is homologous recombination (HR) repair. Defects in HR repair are observed in various cancers, including hereditary breast, and ovarian cancer. Intriguingly, tumor cells with defective HR repair show increased sensitivity to chemotherapeutic reagents, including platinum-containing agents. These observations suggest that HR-proficient tumor cells might be sensitized to chemotherapeutics if HR repair could be therapeutically inactivated. HR repair is an extensively regulated process, which depends strongly on the activity of various other pathways, including cell cycle pathways, protein-control pathways, and growth factor-activated receptor signaling pathways. In this review, we discuss how the mechanistic wiring of HR is controlled by cell-intrinsic or extracellular pathways. Furthermore, we have performed a meta-analysis on available genome-wide RNA interference studies to identify additional pathways that control HR repair. Finally, we discuss how these HR-regulatory pathways may provide therapeutic targets in the context of radio/chemosensitization.

## Introduction

The DNA in each single cell is constantly exposed to a variety of endogenous and exogenous factors that cause DNA lesions, such as UV light and genotoxic chemicals. In addition, normal physiological processes also significantly contribute to generating DNA damage, including cellular metabolism, which produces reactive oxygen species (ROS) as side-products, and DNA replication, which is not an error-free process. To cope with this constant assault on genomic integrity, cells have evolved a complex signaling network called the ‘DNA damage response’ (DDR). The DDR detects DNA lesions, initiates checkpoints that arrest the ongoing cell cycle and in parallel activates dedicated DNA repair pathways (Jackson and Bartek, 2009). Additionally, when the amount of DNA damage exceeds the repair capacity, DDR signaling will clear damaged cells from the proliferative population through senescence or apoptosis.

Defects in DNA repair are frequently observed in cancer and influence the responsiveness of such cancer cells to therapeutic regimens. Particularly, defects in homologous recombination (HR)-mediated repair of DNA breaks caused by hereditary *BRCA1* and *BRCA2* mutations result in increased sensitivity to DNA damaging agents, particularly platinum-based chemotherapeutics (Tan et al., 2008; Alsop et al., 2012). These observations suggest that modulation of HR repair in HR-proficient tumor cells might constitute an effective manner to sensitize cancers for chemotherapy.

Important in this context is the emerging recognition that DNA break repair is under control of many signaling pathways. Also various HR repair-regulatory pathways have been described and a better understanding of how these pathways control HR may provide insight into how HR repair can be inhibited therapeutically to induce chemosensitization. Therefore, we here present an overview of cell-intrinsic or extracellular pathways that control HR repair. Additionally, we performed a meta-analysis on genome-wide siRNA studies to uncover novel HR regulators. Finally, we will elaborate on the potential therapeutic targets within these pathways.

## Repair of DNA Breaks

**FIGURE 1:**
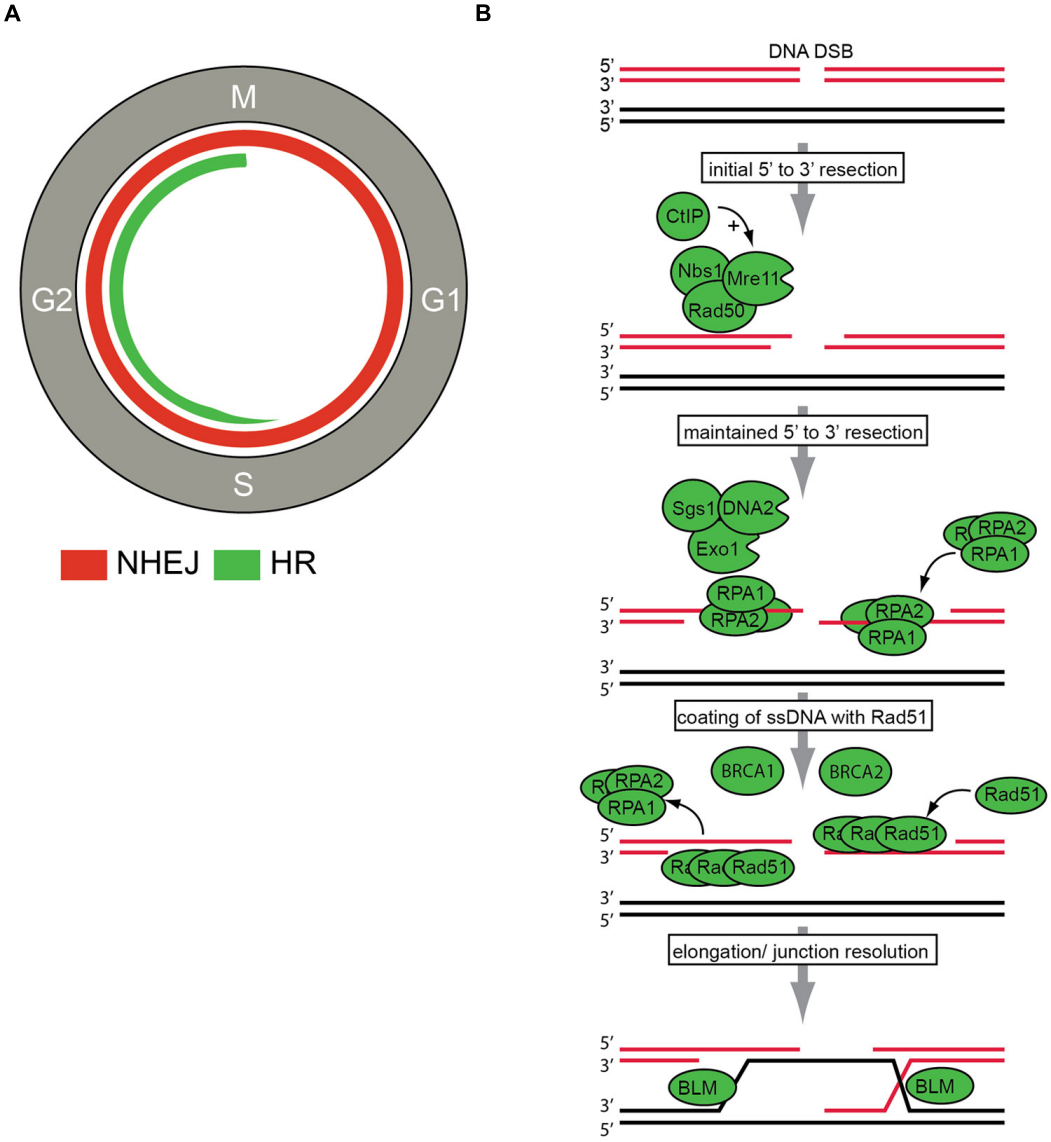
**DNA double strand break (DSBs) repair. (A)** DNA DSBs repair pathways in the context of cell cycle regulation. Non-homologous end joining (NHEJ) can be performed throughout the cell cycle and is indicated with the red line. Homologous recombination (HR) can only be employed in S/G2 phases of the cell cycle and is indicated in green. **(B)** The key steps in HR repair pathway are indicated. After DSB recognition, 5^′^–3^′^ end resection is initiated by the MRN (Mre11, Rad50, Nbs1) complex and CtIP. Subsequently, further resection by the Exo1, DNA2, and Sgs1 proteins is conducted to ensure ‘maintained’ resection. Then, resected DNA-ends are bound by replication protein A (RPA). The actual recombination step within HR repair, termed strand exchange, is executed by the recombinase Rad51. Rad51 replaces RPA to eventually assemble helical nucleoprotein filaments called ‘*presynaptic filaments.*’ This process is facilitated by other HR components, including BRCA1 and BRCA2. Final step of junction resolution is executed by helicases including Bloom syndrome, RecQ helicase-like (BLM) helicase.

Among the various types of DNA lesions, single strand breaks (SSBs) are very prevalent. SSBs can be efficiently repaired through base replacement via base excision repair (BER) or alternatively through removing whole nucleotides via nucleotide excision repair (NER; Caldecott, 2008). Unrepaired SSBs or SSBs that occur during replication can be converted into DNA double strand breaks (DSBs), which are far more toxic. If left unrepaired, only a very limited amount of DNA DSBs is required to cause cell death. Proper repair of these DSBs is therefore crucial for cellular survival. Cells are equipped with two fundamentally different pathways to repair DSBs; non-homologous end-joining (NHEJ) and HR (**Figure 1A**). Non-homologous end-joining can be performed throughout the cell cycle and directly ligates DNA-ends in a non-conservative fashion. Since broken DNA-ends may need cleaning up prior to ligation, NHEJ repair can be mutagenic (a detailed review of NHEJ can be found in Lieber, 2010).

In stark contrast, HR repair utilizes a DNA template for repair with significant sequence homology, and this type of repair is conservative in nature and non-mutagenic (Wyman et al., 2004). Most frequently, sister chromatids are employed for HR, which restricts this type of repair to late S phase and G_2_ phases of the cell cycle, after DNA replication has occurred (Johnson and Jasin, 2000; Krejci et al., 2012). During the highly regulated process of HR, three main phases can be distinguished. Firstly, 3^′^-single-stranded DNA (ssDNA) ends are generated by nucleolytic degradation of the 5^′^-strands. This first step is catalyzed by endonucleases, including the MRN complex (consisting of Mre11, Rad50, and Nbs1). In a second step, the ssDNA-ends are coated by replication protein A (RPA) filaments. In a third step, RPA is replaced by Rad51 in a BRCA1- and BRCA2-dependent process, to ultimately perform the recombinase reaction using a homologous DNA template (**Figure 1B**). More detailed descriptions of HR repair can be found elsewhere (Li and Heyer, 2008; San Filippo et al., 2008). Importantly, HR is not only employed to repair DNA lesions induced by DNA damaging agents, but is also essential for proper chromosome segregation during meiosis. The relevance of HR in these physiological processes is illustrated by its strict requirement during development. Mice lacking key HR genes, such as *Brca1, Brca2,* or *Rad51,* display extensive genetic alterations which lead to early embryonic lethality (Gowen et al., 1996; Hakem et al., 1996; Lim and Hasty, 1996; Ludwig et al., 1997; Sharan et al., 1997; Suzuki et al., 1997). Whereas homozygous inactivation of HR genes is usually embryonic lethal, heterozygous inactivation of for instance *BRCA1* and* BRCA2* does not interfere with cellular viability and rather predisposes to cancer, including breast and ovarian cancer (Futreal et al., 1994; Miki et al., 1994; Wooster et al., 1994; Lancaster et al., 1996). The tumors that develop in individuals with heterozygous* BRCA1/2* mutations invariably lose their second *BRCA1/2* allele, indicating that in certain cancers, the absence of BRCA1/2 is compatible with cellular proliferation. How exactly such tumors cope with their HR defect is currently not fully understood (Elledge and Amon, 2002). What is clear, however, is that these HR-deficient cancers are hypersensitive to various DNA damaging agents, including specific chemotherapeutics (Tan et al., 2008; Alsop et al., 2012). Recent studies have indicated that HR-defective tumors are also exquisitely sensitive to novel agents, such as inhibitors of poly-(ADP-ribose) polymerase (PARP; Bryant et al., 2005; Farmer et al., 2005; Tutt et al., 2010). These insights have prompted the search for cancer-associated mutations in HR genes, to be used for patient stratification for PARP1 inhibitors or other drugs that differentially affect HR-deficient cancers. Additionally, novel components and regulators of the DNA repair machinery are being searched for, to uncover the mechanistic wiring of DNA repair and to uncover potential therapeutic targets for treating cancer.

## Control of HR by the DNA Damage Response

A predominant pathway that controls HR activity is the DDR, which consists of multiple kinase and ubiquitin ligases working in parallel signaling axes to coordinate a cell cycle arrest with DNA repair and induction of apoptosis (Ciccia and Elledge, 2010). Components of the DDR can be functionally classified as (1) sensors of DNA damage, (2) signal transducers, and (3) effectors. Various ‘DNA damage sensor’ complexes exist in order to detect different types of DNA lesions. In the context of DNA breaks, the Mre11/Rad50/Nbs1 (MRN) complex acts as a sensor for DNA DSBs. The MRN complex recruits and activates the upstream DDR kinase ataxia telangiectasia mutated (ATM) (Lee and Paull, 2007). Subsequently, ATM recruits and phosphorylates all MRN members (Gatei et al., 2000; Lim et al., 2000; Zhao et al., 2000; Yuan et al., 2002; Trenz et al., 2006; Linding et al., 2007; Matsuoka et al., 2007). ATM-mediated phosphorylation of these HR components is relevant, as mutational inactivation of these ATM phosphorylation sites prevents the formation of the MRN complex at the sites of damage induced by ionizing radiation (Lim et al., 2000; Zhao et al., 2000), and precludes subsequent cell cycle checkpoint activation and DNA repair (Gatei et al., 2011). MRN/ATM activation consequently leads to the recruitment of additional MRN complexes to the DSB site (Kozlov et al., 2011) and goes along with phosphorylation of other, HR components by ATM, including Brca1 (Cortez et al., 1999; Li et al., 2000) and CtIP (Wang et al., 2013).

Although ATM phosphorylates multiple HR components, it remains unclear to what extent ATM is required for HR. Genetic inactivation of *ATM* in chicken DT40 cells disrupted the formation of irradiation-induced Rad51 and Rad54 foci pointing at impaired HR repair (Morrison et al., 2000; Köcher et al., 2012). However, complete loss of the *Atm* gene in mice did not affect HR capacity in mouse somatic cells in another study (Kass et al., 2013). In contrast, chemical inhibition consistently abrogates HR repair, and points at dominant-negative effects of chemically inhibited ATM (Choi et al., 2010; Kass et al., 2013).

Besides ATM, also ataxia telangiectasia and Rad3 related (ATR) was shown to play a role during HR. In parallel to ATM activation upon DSB formation. The ATR kinase is activated in response to ssDNA, which predominantly occurs at stalled replication forks (Zou and Elledge, 2003). However, ssDNA is also an intermediate product during HR as a result of DSB processing, and leads to ATR activation in response to DSBs (Jazayeri et al., 2006). Later studies showed that ATR activation not merely is a side-product of DNA-end processing, but is actively involved in the process of HR. Specifically, ATR-dependent hyperphosphorylation of CtIP in response to DSBs is required for CtIP accumulation on the chromatin and extension of DNA-end-resection (Peterson et al., 2013). Combined, it appears that ATM is required for an early resection, whereas ATR is responsible for extensive resection and full checkpoint activation. Although the exact roles of ATM and ATR in the regulation of DNA-end-resection during HR are not yet fully understood, the observation that ATR inhibitors block HR repair warrants further investigation of DDR kinases as therapeutic targets to block DNA repair (Prevo et al., 2012).

Besides regulating the recruitment of HR factors to sites of DNA DSBs, also the actual recombination phase of HR repair is regulated by DDR members. ATM, together with c-Abl, regulates the post-translational modification and assembly of Rad51 filaments (Chen et al., 1999). Furthermore, the downstream checkpoint kinase Chk1 was shown to play a role during recombination. Specifically, Chk1 phosphorylates Rad51 at Thr-309 (Sørensen et al., 2005), which consequently facilitates the assembly of Rad51 nucleofilaments by promoting the displacement of RPA with Rad51 and Rad52 (Sleeth et al., 2007). Importantly, Chk1-depletion resulted in abrogation of Rad51 nuclear foci formation in cells exposed to hydroxyurea, illustrating the functional importance of this interaction (Sørensen et al., 2005). In addition, also Chk2 is involved in regulating HR repair, and Chk2-mediated phosphorylation of Brca1 at Ser-988 was shown to be essential for proper recombination repair (Zhang et al., 2004).

Also negative regulators of DNA-end resection, including 53BP1 and Rif1, are phosphorylated by ATM and are recruited to sites of DNA damage in an ATM-dependent fashion (Escribano-Díaz et al., 2013). Specifically, 53BP1 is phosphorylated by ATM at multiple residues and removal of these sites prevents efficient recruitment of 53BP1 to sites of DNA breaks. In turn, Rif1, which is also phosphorylated by ATM, binds 53BP1 in a phospho-dependent manner and is required to block HR to promote NHEJ repair.(Callen et al., 2013; Escribano-Díaz et al., 2013). How exactly DDR signaling can simultaneously promote pro-HR and anti-HR factors is unclear. Very likely, integration of other signaling pathways, including cell cycle kinases, may be important in fine-tuning this response.

Although DDR kinases are clearly important for HR repair, it remains difficult to separate the DNA repair functions from the checkpoint functions of these DDR kinases. For instance, mutation of the multiple ATM phosphorylation sites on Brca1 not only blocks HR repair, but also results in defective intra-S and G_2_/M checkpoint function (Cortez et al., 1999; Xu et al., 1999). Concluding, HR repair appears to be tightly controlled by DDR signaling. However, intense crosstalk and the plethora of proteins that function both in DDR checkpoint signaling as well as in DNA repair, makes it difficult to pinpoint the exact HR regulatory steps in these pathways.

## Cell Cycle Regulation

Homologous recombination repair is tightly coordinated with cell cycle progression, which is in large part governed by cyclin-dependent kinases (CDKs). Yeast studies provided the first notion that HR repair is limited to S and G_2_ phases of the cell cycle and that it is sensitive to chemical CDK inhibition (Aylon et al., 2004). Subsequently, many HR components were shown to be under control of CDKs and that cell cycle kinases, including non-CDKs, control several steps within HR (Aylon et al., 2004; Branzei and Foiani, 2008).

DNA-end resection constitutes the critical decision point to utilize HR or NHEJ for repair of DSBs, and this switch is under prominent control of CDKs. Importantly, if DNA-end resection at sites of DNA breaks has been initiated there is no point of return, because ssDNA cannot be used as a substrate for NHEJ DNA repair (Symington and Gautier, 2011). Clear evidence that break-induced DNA-end resection requires CDK1 was provided in budding yeast (Ira et al., 2004). An important CDK substrate in this process appeared to be Sae2 (in humans called CtIP, encoded by the *RBBP8* gene), which is phosphorylated on Ser-267 in a CDK-dependent fashion (Huertas et al., 2008; Huertas and Jackson, 2009). CDK-mediated phosphorylation of CtIP appeared essential for MRN-mediated DNA-end resection (Limbo et al., 2007; Sartori et al., 2007). In addition to CtIP, also Nbs1 is a CDK target, phosphorylation of which stimulates MRN-dependent end-resection, further underscoring the control of end resection by CDKs (Falck et al., 2012).

Whereas lower eukaryotes have limited numbers of CDKs, mammalian cells have multiple CDKs that can partner with several cyclins (Morgan, 1997), which complicates the analysis of DDR-cell cycle interactions. Nevertheless, initial studies showed that Cdk2 phosphorylation of CtIP stimulates the multimeric interaction between CtIP, Brca1, and the MRN complex (Yu and Chen, 2004; Chen et al., 2008). Specifically, Mre11 is thought to bring Cdk2 and CtIP in close proximity to subsequently promote Cdk2-mediated CtIP phosphorylation (Buis et al., 2012). This interaction has been shown functional, since loss or inhibition of Cdk2 diminishes HR capacity and also results in increased sensitivity to DNA damaging agents (Buis et al., 2012). However, more recent data show that also Cdk1 inactivation decreases HR repair activity (Johnson et al., 2011). These findings may illustrate that different cell types have different CDK activity profiles, and corresponding CDK requirements. Indeed, studies in murine CDK knockout strains illustrated that not one individual CDK but the overall CDKs level highly influences DDR activation in mammalian cells (Murga et al., 2011).

Cyclin-dependent kinases requirements in HR are not restricted to the initiation of DNA-end resection. Even after DNA break resection has been initiated, CDK activity seems to influence HR. Specifically, the stabilization of ssDNA tails is cell cycle-dependent through CDK-mediated phosphorylation of RPA (Anantha et al., 2007). Phosphorylation of the RPA subunit RPA2 at Ser-13 by Cdk1-cyclin B was observed in response to treatment with the chemotherapeutic drug camptothecin. Mutation of these CDK sites in RPA resulted in increased numbers and longer retention of gamma-H2AX and altered cell cycle distribution, and reduced recruitment of other DNA repair factor to sites of DNA damage (Anantha et al., 2007).

Interestingly, recent studies have revealed that not only CDKs but also their bindings partners can influence HR. Two germ-line specific Cdk2 cyclins (A1 and A2) where shown to potentiate HR repair (Müller-Tidow et al., 2004). Although activity of both cyclin A1 and A2 was reported to be required for HR, only cyclin A1 expression was induced by γ-irradiation in a p53-dependent fashion. Additionally, cyclin D1 emerged as a regulator of HR repair (Jirawatnotai et al., 2011). Upon irradiation, Brca2 recruits cyclin D1 to sites of DNA damage, where it directly interacts with Rad51. Moreover, cyclin D1 appears to be essential for Rad51 function, because decreased levels of cyclin D1 severely affected Rad51 recruitment, and consequently resulted in impaired HR. This requirement appeared independent of the canonical cyclin D-binding partners Cdk4 or Cdk6 (Jirawatnotai et al., 2011).

Cyclin-dependent kinases have also been implicated in the regulation of late-stage processes of HR. After recombination has occurred, sister-chromatids can be connected through so-called Holiday junctions, which are resolved by, among others, the Bloom syndrome, RecQ helicase-like (BLM) helicase. Resolution of Holiday junctions, surprisingly, appears to be negatively regulated by CDKs. Notably, Cdk1-dependent phosphorylation of the BLM helicase during mitosis results in dissociation of BLM from the nuclear matrix (Dutertre et al., 2002). However, the functional consequences of this regulation for HR fidelity still remain unclear. Also Brca2 was shown to be negatively regulated by CDKs. Phosphorylation of Brca2 at Ser-3291 within its C-terminal domain prevents the Brca2–Rad51 interaction and thus impairs Rad51-mediated foci formation (Esashi et al., 2005). The phosphorylation of Brca2 at Ser-3291 appears to depend on Cdk1, since chemical inhibition of Cdk1 activity diminishes Brca2-Ser-3291 phosphorylation (Krajewska et al., 2013). In line with Cdk1 activity being most prevalent during mitosis, this mechanism may functionally restrict HR to those phases of the cell cycle when sister chromatids are available for HR repair. Notably, mitotic inactivation of HR can be exploited using Wee1 inhibitors that can aberrantly activate Cdk1. This results in a block in HR repair, underscoring that CDKs not only activate HR during S-phase, but also block HR during mitosis.

**FIGURE 2:**
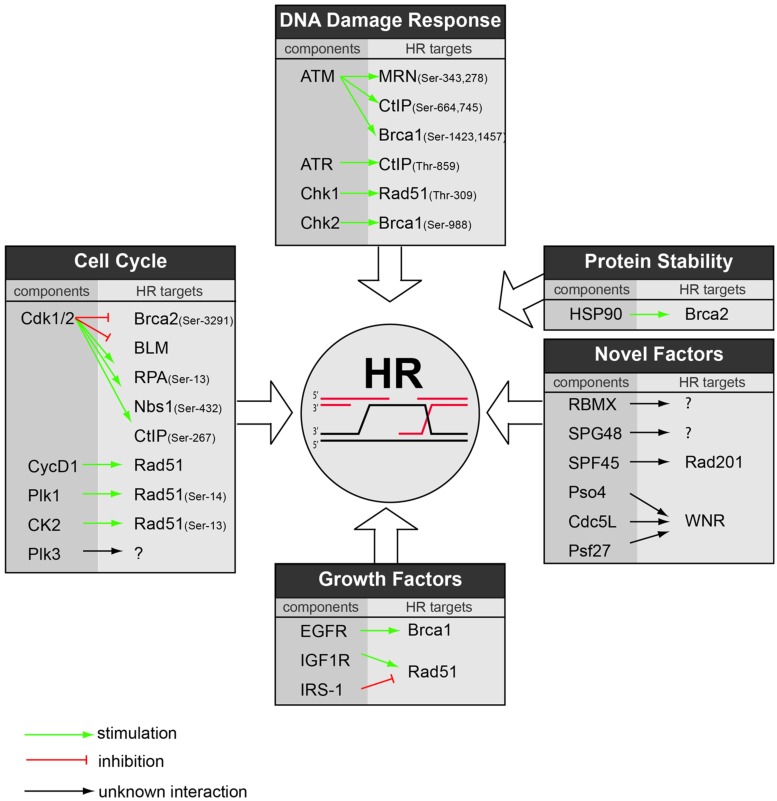
**Regulators of HR repair as potential therapeutic targets.** Based on literature and the GSEA performed in this report, an overview is provided on the various cellular pathways that regulate HR repair. For each pathway, the responsible component is highlighted along with its substrate within HR. Green arrows indicate stimulatory interactions, red bars indicate inhibitory interactions, and black arrows indicate interactions with unclear effect.

Beyond the CDK-mediated regulation of HR, also other cell cycle kinases were shown to influence HR fidelity. Polo-like kinase 1 (Plk1), for instance, which is required for mitotic entry and mitotic progression (van Vugt and Medema, 2005) was shown to regulate HR. In concert with the cell cycle kinase Casein kinase-2 (CK2), Plk1 phosphorylates Rad51 at Ser-14, which is required for the Rad51 filament formation (Yata et al., 2012). Subsequently, CK2 phosphorylates Rad51 at Thr-13 to enhance the interaction between Rad51 and Nbs1 and to facilitate Rad51 recruitment to sites of DNA damage (Yata et al., 2012). In addition to a direct regulation of Rad51, Plk1 binds, and phosphorylates Brca2 (Lin et al., 2003). This interaction appears to be abrogated after DNA damage induction, suggesting that Plk1 may also negatively influence HR repair. Additionally, Plk3 was implicated in the regulation of DNA break repair through modification of CtIP (Barton et al., 2014). In addition, cells lacking Plk3 were shown to be sensitive to PARP inhibitors, which suggests a role for Plk3 in the HR repair (Turner et al., 2008). However, the exact role for Plk3 within the HR machinery needs to be elucidated. Combined, these data imply that cell cycle kinases other than CDKs are required to properly activate HR repair as well as control its silencing when appropriate (**Figure 2**).

## Protein Stability Control

As for all cellular pathways, correct protein folding is essential for proper execution of DNA repair. Protein folding is mediated by so-called ‘protein-stability control’ pathways, controlled by heat-shock protein (HSP) family members (Lindquist and Craig, 1988) Among their many client proteins, several cell cycle control, and DNA repair components appear to be under control of these molecular chaperons. Specifically, Hsp90 appears to control the stabilization, folding, and activation of key HR repair signaling proteins. Most prominently, inhibition of Hsp90 using 17-AAG resulted in Brca2 destabilization (Noguchi et al., 2006; Dungey et al., 2009; **Figure 2**). In line with blocking Brca2 function, Hsp90 inhibition delayed Rad51 filament formation (Noguchi et al., 2006) and resulted in radiosensitization, which was enhanced by the addition of PARP inhibitors (Dungey et al., 2009). Later studies using the more potent Hsp90 inhibitor NVP-AUY922 confirmed these HR defects and described potent radiosensitiziting effects *in vivo* (Zaidi et al., 2012).

The observations that Brca2 depends heavily on protein-stability chaperons have initiated investigations to see whether Brca2 could be destabilized by mild hyperthermia. Indeed, Brca2 is efficiently but transiently destabilized by a short-term cellular hyperthermia (41–42,5^∘^C; Krawczyk et al., 2011). As a consequence, hyperthermia blocked the recruitment of Rad51 to sites of DNA damage and led to impaired HR (Krawczyk et al., 2011). These HR defects coincided with radiosensitization and increased sensitivity for PARP inhibitors *in vitro* and *in vivo* (Krawczyk et al., 2011). Clearly, these findings offer clinical opportunities, since it allows local induction of HR deficiency, which could be used to sensitize tumors for concomitant radiotherapy and PARP inhibitor treatment (Eppink et al., 2012). In addition, the observed Brca2 destabilization offers an appealing explanation for the earlier observed radio- and chemosensitizing effects of hyperthermia, both pre-clinically and clinically (Overgaard et al., 1995; Vernon et al., 1996; Sneed et al., 1998; van der Zee et al., 2000). However, it should be noted that defective HR through Brca2 inactivation does not explain the entire radiosensitizing effect of hyperthermia. Using isogenic cell lines with defects in various repair pathways, HR only partially contributed (Kampinga et al., 2004). In addition, effects of hyperthermia were observed in all cycle phases, which does not support the cell cycle-restricted action of HR repair, suggesting additional targets for hyperthermia in DNA repair (Dewey et al., 1978; Kim et al., 1978). Summarizing, current data support the protein-stability machinery as a feasible therapeutic target to decrease HR capacity, either using Hsp90 inhibitors or through mild hyperthermia. Further, these results warrant clinical studies to combine these approaches with genotoxic therapies that are especially effective in HR-deficient cancers, including PARP inhibitors and platinum-containing chemotherapeutics.

In addition to control of HR DNA repair by HSPs, multiple other enzymes have been to control the stability of DNA repair components. Classically, modification of proteins with ubiquitin has been linked to protein-stability control (Hershko and Ciechanover, 1998). Within the DDR, however, ubiquitilation (as well as SUMOylation) have been shown primarily with activation and protein complex formation (Jackson and Durocher, 2013). However, recently the key HR component CtIP was shown to be ubiquitilated by the APC/C-Cdh1 in a cell cycle and DNA damage-dependent fashion (Lafranchi et al., 2014). Whether the APC/C-Cdh1 controls other DDR proteins upon DNA damage, and whether this affects DNA repair needs further investigation.

## Regulation of HR Repair by Growth Factor Receptor Signaling

Growth hormone receptor pathways encompass multiple signaling cascades, controlling many cellular processes including proliferation, cellular survival, and migration. These pathways, including the epidermal growth factor receptor (EGFR) pathway, are frequently hyperactivated in cancers through mutation or amplification and constitute so-called ‘oncogenic drivers’ (Sharma et al., 2007). However, part of their oncogenic potential may also be explained by promoting DNA repair. Indeed, growth hormone receptor signaling contributes to increased resistance to radio- or chemotherapy, which likely is related to modulation of DNA repair (Mukherjee et al., 2009). With growth factor receptors being oncogenic drivers, multiple therapeutics have been clinically developed to target growth factor receptors (including antibodies and small molecule inhibitors targeting the EGFR, HER2, and IGF1R). When tested in combination with chemo-radiotherapy, these agents appear to improve responses to radio- and chemotherapy in several cancer types (Huang and Harari, 2000; Bonner et al., 2006). Furthermore, small molecule tyrosine inhibitors that ablate kinase activity of oncogenic variants of these receptors (including erlotinib and gefitinib) have clinical benefit in combination with chemotherapeutics in multiple pre-clinical and clinical studies (Moyer et al., 1997; Knight et al., 2004; Mellinghoff et al., 2005; Quatrale et al., 2011).

Since growth factor receptors control various downstream pathways related to growth and survival, it has remained difficult to pinpoint the influence of growth factor receptor signaling on DNA repair. In addition, it is not completely clear through which mechanism(s) growth factor receptor signaling influences DNA repair, and which DNA repair subtypes are actually modulated by such pathways. In the context of DNA DSB repair, both NHEJ as well as HR were shown to be under control of growth hormone receptor-mediated signaling. Treatment of cells with EGF was shown to increase levels of NHEJ as well as HR (Golding et al., 2009; Myllynen et al., 2011). In the context of promoting NHEJ, the EGFR was reported to associate with the catalytic subunit of DNA-PK, an essential NHEJ component (Liccardi et al., 2011). Additionally, nuclear localization of the EGFR required for DNA repair stimulation, occurs through its interaction with DNA-PK (Liccardi et al., 2011). Additionally, stimulation of the EGFR or the insulin-like growth factor receptor 1 (IGF1R) also elevates levels of HR repair (Golding et al., 2009; Myllynen et al., 2011). Concerning the role of the EGFR in HR repair, it was shown that EGFR activity is required for Brca1 localization to the nucleus (Li et al., 2008). Consequently, blocking the EGFR using erlotinib prevents nuclear Brca1 localization, interferes with Rad51 recruitment to sites of DNA damage and attenuates HR repair (Li et al., 2008). Surprisingly, the role of the IGF1R in HR repair appeared to be mechanistically distinct. IGF1R signaling promotes cellular trafficking of Rad51 through a direct interaction between the insulin receptor substrate-1 (IRS-1), which is recruited to sites of DNA lesions in response to DNA damage (Trojanek et al., 2003). In line with this observation, blocking IGF1R function through deletion of the *Igf1r* gene in mice, or IGF1R depletion ablates IRS-1 phosphorylation, precludes Rad51 translocation to the nucleus and eventually impairs HR repair (Trojanek et al., 2003). Also estrogen-mediated phosphorylation of IRS-1 by the estrogen receptor beta (ERβ) affects HR repair (Urbanska et al., 2009). In contrast to insulin receptor signaling and EGFR signaling, surprisingly, ER signaling negatively impacts the Rad51 function, and inhibition of ERβ-mediated IRS-1 translocation to the nucleus significantly improved DNA repair fidelity and prevented genomic instability (Urbanska et al., 2009). Collectively, multiple growth factor or hormone receptors impact on DNA repair through direct or indirect interactions with DNA repair proteins, albeit that different receptors may have opposite effects in regulating DNA repair.

Since part of the synergistic effects of combined radio/chemotherapy with targeting growth-factor-activated receptors may be explained by interfering with DNA repair, a synthetic lethal context with agents such as PARP inhibitors may be created. Early preclinical evidence indeed underscores this notion, since EGFR inhibition with lapatinib sensitized breast cancer cells to the PARP inhibitor ABT-888 *in vitro* (Nowsheen et al., 2012). In addition, therapeutic targeting of the PI3 kinase, which operates downstream of the EGFR and IGF1R efficiency blocked HR repair through down regulation of both Brca1 and Brca2 and sensitized cells for PARP inhibition (Ibrahim et al., 2012). Concluding, HR DNA repair is not just a cell-intrinsic repair mechanism. Many pathways, including growth factor-activated pathways, were shown to regulate HR, providing a rationale for combined inhibition of growth factor activated pathways with DNA damaging agents.

## Novel Regulators of Homologous Recombination

**FIGURE 3:**
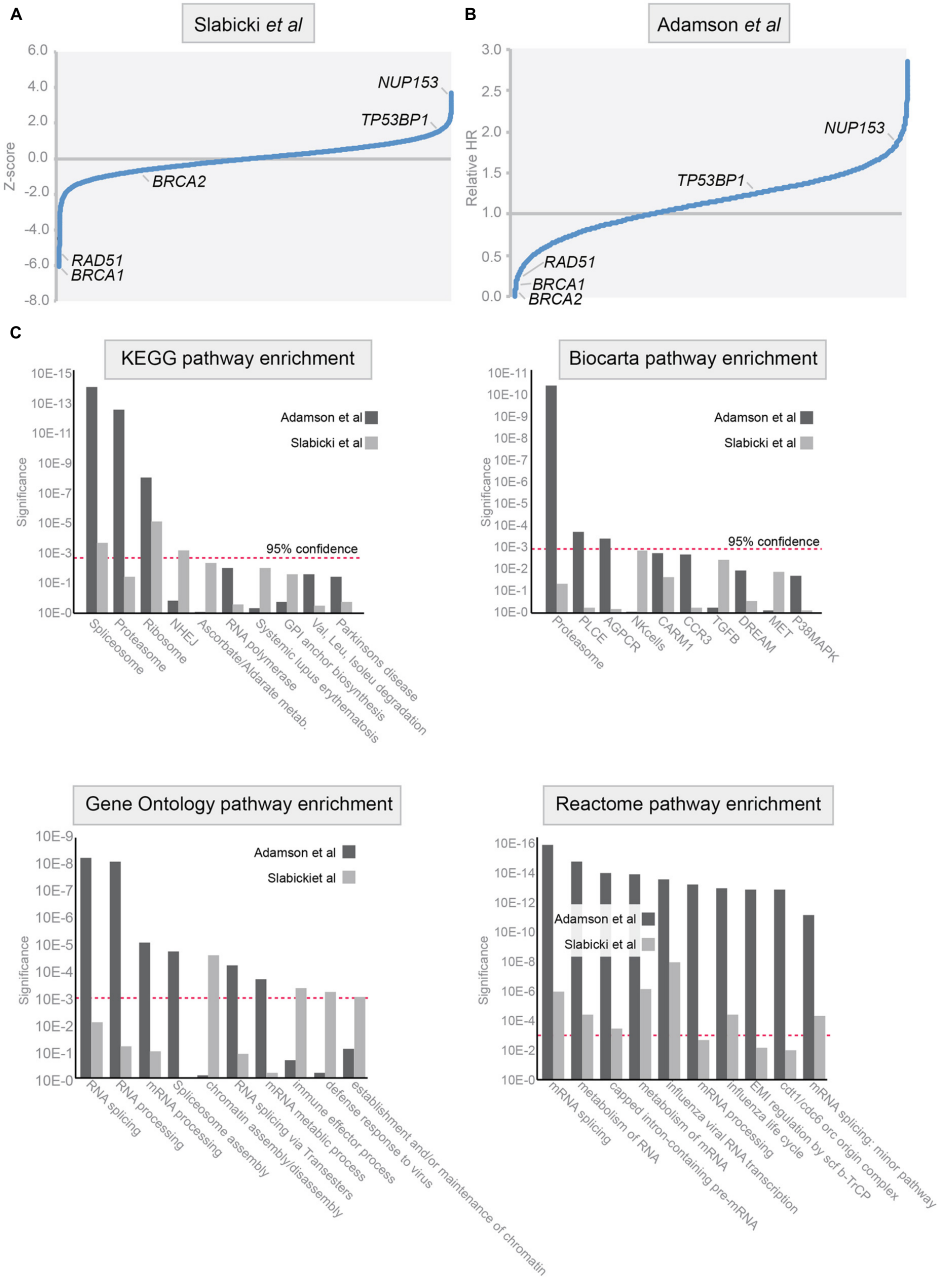
**Pathways involved in homologous recombination repair of DSBs. (A,B)** Analysis of the data from genome-wide siRNA screens. Data were adapted from published studies (Słabicki et al., 2010, **A**) and (Adamson et al., 2012, **B**). In these studies HR efficiency was assessed using DR-GFP assay in HeLa cells (left) and in DR-U2OS cells (right). Relative HR scores in *Z*-score (left panel) and ‘Relative HR score’ (right panel) are indicated for genes with an established role in HR: *BRCA1, BRCA2, TP53BP1, NUP153,* and *RAD51*. **(C)** Gene-Set Enrichment Analysis (GSEA) was performed on the data presented in **(A,B)**. Four pathway databases were used for enrichment analysis: KEGG, Biocarta, Reactome, and GeneOntology. Results of the GSEA on the dataset by Słabicki et al. (2010) are represented in light gray, and results on the dataset by Adamson et al. (2012) are represented in dark gray. Red dashed line indicates 95% confidence interval.

The development of fluorescence-based reporter systems to read out HR efficiency (Pierce et al., 1999) has enabled high-throughput microscopy studies to uncover novel regulators of HR in mammalian cells. Two important studies have taken a genome-wide approach to identify genes that are required for HR repair (Słabicki et al., 2010; Adamson et al., 2012). Many of the identified genes from these studies are highly conserved and also appear to be essential for HR in single-cell organisms such as yeast, including *BRCA1*, *BRCA2,* and *RAD51* (McKinney et al., 2013). In addition to these well-known HR components, novel HR regulators were identified. Notably, genes that control post-transcriptional processing of RNA, including mRNA splicing, where found to control HR repair (Adamson et al., 2012). For instance, depletion of the RNA binding protein RBMX led to diminished Brca2 levels and a consequent failure to recruit Rad51 to sites of DNA damage. Additionally, a putative helicase SPG48 is required for HR repair, although it remains mechanistically unclear which step of HR it controls (Słabicki et al., 2010). The availability of two independent genome-wide siRNA screens for genes that regulate HR allowed us to compare these data sets and identify common pathways and genes. To this end, we applied Gene-Set Enrichment Analysis (GSEA; Subramanian et al., 2005) to uncover additional pathways that modify HR repair, of which targeting could have therapeutic potential (**Figures 3A,B**). Four well-known pathway databases were used for enrichment analysis, KEGG, Biocarta, Reactome, and GeneOntology (**Figure 3C**).

The most striking enrichment was identified in genes that participate in proteasome or RNA biology. These findings match earlier reports demonstrating that proteasome inhibition sensitizes tumor cells to DNA damaging agents, including crosslinking agents, and radiation (Pajonk et al., 2000). Initially, these effects were explained by proteasome-mediated control of pro-apoptotic p53 signaling (Vaziri et al., 2005). Thereafter, a more direct role for the proteasome in controlling DNA repair was identified. Inhibition of the proteasome using MG132 or bortezomib, or genetic inactivation of proteasome components blocked the recruitment of DNA repair components FancD2, Brca1, and Rad51, whereas upstream the DDR signaling components H2AX and Mdc1 appeared unaffected (Jacquemont and Taniguchi, 2007; Cron et al., 2013). Due to defective recruitment of these HR factors, inhibitors of the proteasome suppress homologous DNA recombination in mammalian cells (Murakawa et al., 2007). In line with this notion, proteasome inhibition using bortezomib was shown to prevent repair of PARP-inhibitor induced DNA breaks (Neri et al., 2011). Importantly, combined treatment with bortezomib and the PARP inhibitor ABT-888 resulted in sustained levels of H2AX, with defective recruitment of HR repair components leading to enhanced killing of tumor cells. Combined, these results show that the proteasome is involved in HR repair and that therapeutic targeting of the proteasome, using for instance bortezomib, can be used to induce ‘BRCAness’ in tumor cells. Also, the fact that genome-wide *in vitro* assays identified the proteasome as a system that controls HR repair argues that other HR-controlling pathways may also be uncovered using these approaches.

Among the enriched pathways involved in HR repair, RNA processing was highly abundant (**Figure 3C**), as is matched by the recent identification of RNA-modifying enzymes as regulators of HR (Adamson et al., 2012). In mammalian cells, not much data exist that mechanistically link RNA to HR repair. However, some data from model organisms have provided evidence that RNA processing is involved in DNA repair. Using genetic analysis in *Drosophila*, the RNA splice factor SPF45 was shown to combine a function in RNA splicing and protection against DNA damage caused by MMS exposure (Chaouki and Salz, 2006). Notably, mutations that abolish the RNA splicing function also fail to protect against DNA damage-induced toxicity. Mechanistically, SPF45 appears to interact with Rad201, a member of the RecQ/Rad51 family (Chaouki and Salz, 2006). An unbiased proteomic screening in budding yeast underscored the link between RNA metabolism and DNA repair, for instance by identifying the splicing factor PRP19, as being involved in the DDR (Smolka et al., 2007). In human cells, a mRNA splicing complex consisting of Pso4, Cdc5L, and Psf27 was found to interact with the WNR DNA helicase and was identified to be required for interstrand cross-link repair (Zhang et al., 2005; **Figure 2**). Additionally, Dicer and Drosha RNA products produced from sites of DNA damage where shown to be required for proper DDR signaling (Francia et al., 2012). Again, large-scale proteomic analysis confirmed this notion, and identified multiple factors involved in RNA metabolism, illustrating the intricate connection between RNA splicing and DNA repair (Matsuoka et al., 2007). Part of this relationship can be explained by the observation that uncontrolled mRNA maturation disturbs the DNA-RNA interaction and have deleterious effects on genomic stability (Montecucco and Biamonti, 2013).

## Targeting HR-Deficient Tumors Clinically

In oncology, many radio- and/or chemotherapeutic regimens are used in daily practice, which induce high levels of DNA damage directly or indirectly. These therapeutic regimens often induce interstrand DNA crosslinks and DNA DSBs, of which accumulation is very cytotoxic and requires HR for faithful repair. Consequently, tumors in which HR repair is compromised due to mutations or epigenetic silencing of HR repair genes are generally more sensitive to specific DNA damage-inducing factors. Extensive *in vitro* and *in vivo* preclinical studies provided compelling evidence that HR defects are causally related to the vulnerability of such cancer cells to certain DNA damaging chemotherapeutics and radiotherapy. Studies comparing various neo-adjuvant chemotherapeutic regimens in *BRCA1* mutant breast cancers, found that highest response rates were observed with neo-adjuvant cisplatin chemotherapy (Byrski et al., 2010), which increased progression free survival (Byrski et al., 2008). In analogy, responses of ovarian cancer lines to cisplatin were also influenced by their *BRCA1/2* mutation status as well as the status of related HR genes (Taniguchi et al., 2003). These data indicate that inactivation of the HR pathway, either through germ-line or somatic *BRCA1/2* inactivation is linked to increased sensitivity to DNA damaging therapeutics, notably platinum-based agents.

The fact that DNA repair defects through cancer-associated mutations lead to specific vulnerabilities is exploited in synthetic lethal approaches: mutation or inhibition of two separate pathways leads to cell death, whereas loss of function in either one of these pathways does not affect viability. The prototypical synthetic lethal interaction was described for the *BRCA1* and *BRCA2* mutations, which are synthetic lethal with loss of PARP-1 (Bryant et al., 2005; Farmer et al., 2005). Mechanistically, inhibition of PARP1 blocks BER and leads to accumulation of SSBs, which are converted to DSBs in replicating cells (Bryant et al., 2005; Farmer et al., 2005). These DSBs cannot be properly repaired due to the HR defect in *BRCA1/2* mutant cells, which results in cytotoxicity Recent studies provided evidence that PARP is additionally required for restarting of stalled replication forks (Bryant et al., 2009; Petermann et al., 2010). PARP1 accumulates at stalled replication forks and recruits Mre11 to catalyze DNA-end processing for replication restart and recombination. As a consequence, PARP inhibition also results in replication forks collapse, which again leads to accumulation of toxic DNA structures in HR-deficient cells (Schlacher et al., 2011). The finding that *BRCA1/2* mutant cells are selectively sensitive to PARP inhibition constituted the starting point for several clinical trials and the clinical development of various PARP inhibitors. In 2005, the first phase I clinical study investigating a PARP inhibitor (Olaparib, AZD2281) demonstrated that more than 90% of PARP enzymatic activity could be inhibited, which was well tolerated and did not increase toxicity in the *BRCA1/2* mutation carriers group (Fong et al., 2009). More importantly, this study showed clinical benefit of PARP inhibition in patients with *BRCA1/2* mutations (Fong et al., 2009). This early success prompted the clinical development of various PARP inhibitors as single agents or as part of combined treatment with DNA damaging agents in phase II clinical trials (Audeh et al., 2010; Tutt et al., 2010). In addition the reported beneficial effect of PARP inhibition in breast and ovarian patients with *BRCA1/2* mutations boosted the interest in this type of therapies for other tumors types, including colon cancers, prostate cancer, and gastric cancer (Barreto-Andrade et al., 2011; Sebastian de Bono et al., 2011; Davidson et al., 2013). Results of preformed or ongoing clinical trials with PARP inhibitors are therefore eagerly awaited.

The preclinical studies and early clinical studies raised high potential for therapeutic use of PARP1 inhibitors, but unfortunately PARP inhibitors have not yet delivered the clinical success that preclinical studies promised. Several findings can be attributed to these discrepancies.

Firstly, it appears difficult to effectively select patients for PARP1 inhibitor treatment. The most straightforward strategy to select patients is to obtain the mutation status of *BRCA1/2* in cancer specimens. However, *BRCA1* or *BRCA2* are not only inactivated through gene mutation, also DNA hypermethylation of the genes is frequently reported for several cancers (Esteller et al., 2000; Rice et al., 2000; Cancer Genome Atlas Research Network, 2011). Patients with hypermethylated *BRCA1/2* genes may benefit from PARP1 inhibitors, but may be missed when only *BRCA1/2* mutations status is analyzed. In contrast, when selection criteria are not sufficiently strict, effects of PARP inhibitors may be missed. For example, some studies included all TNBCs, whereas only a subset of these patients may harbor HR defects, and may therefore have clinical benefit from PARP inhibitors (Gelmon et al., 2011). A straightforward, but labor-intense solution to successfully implement PARP inhibitors is to functionally assess HR efficiency in fresh tumor biopsies (Willers et al., 2009). Alternatively, measuring consequences of defective HR, such as genome-wide copy number variation analysis using aCGH may identify tumors with defective HR (Vollebergh et al., 2011). Besides selecting *BRCA1/2* mutant tumors, also mutation of other HR genes results in cancers with similar characteristics, including *PALB2* (Rahman et al., 2007) or *RAD51C* (Clague et al., 2011). Mutation of these genes appeared to result in PARP1 inhibitor sensitivity (Loveday et al., 2012; Min et al., 2013). Also novel regulators of HR may be important in this respect, such as the negative regulator of Brca1 called ID4 (Turner et al., 2007). Whether ID4 mutations or mutations in other novel HR components are frequently found in cancers needs to be studies. Clearly, these observations indicate that there is a strong need to reliably identify cancers with defective HR repair, in order to stratify patient for therapies that target HR-deficient cancers, including PARP1 inhibitors.

Secondly, not all PARP inhibitors appear to be very efficient PARP inhibitors *in vivo*. Moreover, some PARP inhibitors have additional effects. For instance, iniparib (BSI-201) also inhibits other enzymes, including GAPDH (Bauer et al., 2002). It is therefore difficult to verify whether observed clinical benefit in phase II study was achieved exclusively due to PARP inhibition.

A third complicating factor in developing PARP inhibitors for HR-deficient cancers, is the recent observation that secondary mutations may dramatically alter the HR defect of *BRCA1/2* mutation cancers. These secondary mutations can be sub-classified into two categories. The first category consists of intragenic secondary mutations in affected *BRCA1* or *BRCA2* alleles that have been described to restore their reading frame (Sakai et al., 2008; Swisher et al., 2008) and result in resistance to cisplatin. The second category involves secondary mutations in other genes, which have been shown to reverse a HR defect. Mutation of *TP53BP1* for instance, reverses the HR defect in *BRCA1* mutant cancers and render these cancers resistant to PARP1 inhibition. More recently, Rif1 was shown to counteract Brca1 function, and *Rif1* mutations could rescue the genomic instability of mouse *Brca1^-^*^/-^ cells (Bouwman et al., 2010; Chapman et al., 2012; Di Virgilio et al., 2013; Escribano-Díaz et al., 2013; Feng et al., 2013; Zimmermann et al., 2013). Whether *Rif1* mutations also account for therapy resistance in *BRCA1* mutant cancers remains to be tested. What is clear is that *BRCA1/2* mutations do not per se reflect a defect in HR, and that functional testing of HR capacity may be required to reliably classify the DNA repair defect in cancers.

## Future Perspectives

If HR function could be locally inhibited in cancer cells, this would allow exploitation of the enhanced sensitivity for platinum-containing chemotherapeutics, radiotherapy, or PARP inhibition. The most straightforward approach would be to directly target HR components. The identification of druggable HR genes is therefore actively being pursued. One approach is to chemically inhibit Rad51, the most downstream HR component. Recent studies making use of high-throughput screens have identified chemical Rad51 inhibitors, which increased the sensitivity of glioblastoma cells to alkylating agents (Quiros et al., 2011), and were shown to sensitize various human cancer cells to DNA crosslinking agents, including mitomycin C (Budke et al., 2012).

In addition to directly targeting HR components, the reports described in this review show that modulation of regulatory pathways controlling HR components may be useful as well to achieve an HR-deficient phenotype and thereby sensitize tumor cells to DNA damaging agents (an overview of cellular pathways that regulate HR repair is presented in **Figure 2**). This has been elegantly shown in pre-clinical studies using cell cycle modulators, hyperthermia, DDR inhibitors and Hsp90 inhibitors. Novel approaches, including genome-wide siRNA screens and proteomic interaction maps, may add novel regulators to this growing list of potential therapeutic targets that control HR and warrant translation of these novel targets to uncover their therapeutic potential.

## Conflict of Interest Statement

The authors declare that the research was conducted in the absence of any commercial or financial relationships that could be construed as a potential conflict of interest.
